# Hypocalcemia Revisited: Thinking Outside the Box!

**DOI:** 10.7759/cureus.95697

**Published:** 2025-10-29

**Authors:** Hayder Al-Khalafawi, Ali Akber Rajani, Darshi Sivakumaran

**Affiliations:** 1 General Internal Medicine, Kingston and Richmond NHS Foundation Trust, London, GBR; 2 Internal Medicine, London North West University Healthcare NHS Trust, London, GBR

**Keywords:** genetic hypocalcemia, hypocalcemia causes, pseudohypoparathyroidism, pseudohypoparathyroidism type 1b, resistant hypocalcemia

## Abstract

This case report details the presentation of a 62-year-old man with a background of hypothyroidism, hypertension, and chronic kidney disease (CKD) who experienced fatigue with acute-on-chronic hypocalcemia. Despite receiving oral calcium carbonate with vitamin D3 (cholecalciferol) supplementation in primary care, persistent hypocalcemia in the absence of significant renal impairment and a positive response to alfacalcidol (1α-hydroxycholecalciferol) prompted further investigation into genetic causes. A final diagnosis of pseudohypoparathyroidism type 1b (PHP1b) was confirmed through genetic studies. In cases of pseudohypoparathyroidism (PHP), appropriate monitoring and timely management are essential for patient safety and favorable outcomes. This case explores the systematic approach required in the assessment of chronic hypocalcemia to discern aetiology among a wider range of differentials.

## Introduction

Hypocalcemia, defined as a total serum calcium level less than 2.2 mmol/L [1], is a frequently encountered biochemical abnormality in clinical practice. Severe hypocalcemia can necessitate admission, while mild or moderate chronic hypocalcemia is often asymptomatic. The causes of hypocalcemia can be broadly divided into either parathyroid hormone (PTH)-mediated or non-PTH-mediated (e.g., vitamin D deficiency) disorders [2]. This case spotlights a dysfunction in the PTH axis and the structured approach required in the assessment of a non-specific finding of hypocalcemia to help discern an underlying rare aetiology. Systematic assessment enables the early recognition and diagnosis of less common causes such as pseudohypoparathyroidism (PHP).

The prevalence of PHP is uncertain; however, epidemiological studies from Denmark and Japan report estimates near 1.2 cases per 100,000 [3]. PHP is characterized by peripheral tissue-specific resistance to PTH secondary to underlying stimulatory G-protein (Gsα) dysfunction. Adult-onset pseudohypoparathyroidism type 1b (PHP1b) is rare and easily overlooked. Biochemically, PTH resistance profiles may feature hypocalcemia, hyperphosphatemia, and high serum PTH levels [2]. Clinically, PHP diagnosis can be aided by examination with the pseudohypoparathyroidism type 1a (PHP1a), pseudopseudohypoparathyroidism (PPHP), and pseudohypoparathyroidism type 1c (PHP1c) subtypes including features such as short stature, brachydactyly of the third and fourth metacarpals ("knuckle-knuckle-dimple-dimple" sign), and obesity, a constellation of signs termed as Albright hereditary osteodystrophy (AHO) [4]. More difficult-to-diagnose subtypes, including PHP1b, may lack clear examinable findings and rely on a focused history, assisted further by genetic testing.

PHP serves as a valuable example in understanding hormone resistance syndromes, which may result in complications such as hypocalcemia, skeletal abnormalities, cognitive and cardiac issues, and other endocrine dysfunctions (e.g., thyroid-stimulating hormone (TSH) resistance) [5]. This paper highlights one presentation of adult-onset PHP1b and aims to improve awareness among clinicians to enhance diagnostic accuracy in presentations to primary care and outpatient settings.

## Case presentation

A 62-year-old Caucasian man with a background of chronic kidney disease (CKD) from 2022, most likely due to high-pressure chronic urinary retention, hypertension, and primary hypothyroidism, presented with worsening fatigue. Notably, hypocalcemia had been documented in 2015 but was not monitored subsequently.

In August 2023, blood tests arranged by his general practitioner (GP) revealed severe hypocalcemia with an adjusted calcium of 1.75 mmol/L (reference range: 2.2-2.6 mmol/L); his phosphate was elevated at 1.58 mmol/L (reference range: 0.8-1.5 mmol/L), while renal function remained stable with a creatinine of 145 µmol/L (reference range: 60-106 µmol/L) and an estimated glomerular filtration rate (eGFR) of 44 mL/min/1.73 m². Reviewing historical results, his adjusted calcium had been similarly low in September and December 2015, although his eGFR was >60 mL/min/1.73 m² at that time and his vitamin D levels were replete at 86 nmol/L (reference range: 50-174 nmol/L). His PTH level in December 2015 was elevated at 15.7 pmol/L (reference range: 1.1-6.9 pmol/L).

Following his blood test in August 2023, he was referred to same-day emergency care (SDEC) at a district general hospital. Physical examination was unremarkable, and Chvostek's sign was negative. His medications included amlodipine 5 mg, atorvastatin 20 mg, and levothyroxine 125 µg daily. He was advised to take oral calcium supplements for three days, with follow-up planned to reassess calcium levels. SDEC contacted him the following day, but he had not yet started calcium supplementation and was awaiting further blood tests and advice from his GP.

In November 2023, repeat blood tests showed persistent hypocalcemia with an adjusted calcium of 1.8 mmol/L (reference range: 2.2-2.6 mmol/L). He was re-referred to SDEC. His PTH from August 2023 was elevated at 14.5 pmol/L (reference range: 1.1-6.9 pmol/L), and his vitamin D levels remained normal at 92 nmol/L in August and 101 nmol/L in November (reference range: 50-174 nmol/L). Magnesium was also normal at 0.84 mmol/L (reference range: 0.7-1 mmol/L).

He was advised to increase Adcal-D3 (cholecalciferol-calcium carbonate) from twice daily to three times daily and was referred to endocrinology for outpatient follow-up. In February 2024, he was reviewed in the endocrinology department. All available results, including bone profile, PTH, vitamin D, magnesium, and renal function, were examined, including historical data from 2015. A focused history revealed his main concerns were lethargy and daytime somnolence. He had been diagnosed with primary hypothyroidism in his 30s. There was no family history of hypocalcemia. He had short stature relative to his male siblings but no history of obesity or shortened digits. His body mass index (BMI) at this assessment was 24.6 kg/m^2^. His skin examination revealed small seborrheic warts and areas of brown pigmentation, but no subcutaneous calcification. He had a round face. Overall, there were no definitive features of AHO.

His renal function was not impaired enough to explain PTH resistance, and similar biochemical findings had been present 15 years earlier when his renal function was normal. PHP was therefore suspected. A genetic study was requested, and Adcal-D3 was replaced with alfacalcidol (an active vitamin D analogue) at 250 ng daily. The dose was titrated to target an adjusted calcium of 2.1 mmol/L (reference range: 2.2-2.6 mmol/L), and he was advised to follow a low-phosphate diet.

His serum prolactin level was low at 72 mIU/L (reference range: 86-324 mIU/L), which can be associated with PHP, as can hypothyroidism. His follicle-stimulating hormone (FSH) was mildly elevated at 13 IU/L (reference range: 2-12 IU/L), suggesting some possible impairment of spermatogenesis, although this was not considered clinically significant given the patient's age. He had normal luteinizing hormone (LH) and free testosterone levels at 7.9 IU/L (reference range: 2-9 IU/L) and 0.265 nmol/L (reference range: 0.17-0.66 nmol/L), respectively. His insulin-like growth factor 1 (IGF-1) was also within the normal range at 14.8 nmol/L (reference range: 7.5-24.2 nmol/L).

Genetic testing confirmed PHP1b, with a heterozygous deletion of exons 5-7 in the STX16 gene and complete loss of maternal methylation at the GNAS A/B:TSS differentially methylated region. This indicated the deletion was maternally inherited. The report concluded a 50% chance of transmission to offspring, who may not manifest the condition, though future generations could be affected.

The diagnosis was explained to the patient, and he was referred to a tertiary hospital's clinical genetics service. Follow-up blood tests showed significant improvement in adjusted calcium (2.15 mmol/L; reference range: 2.2-2.6 mmol/L) and phosphate (1.34 mmol/L; reference range: 0.81-1.5 mmol/L) on alfacalcidol 750 ng daily and a low-phosphate diet. A 24-hour urine calcium output was measured on this regimen in October 2024 and was satisfactory at 3.54 mmol/24 hr (reference range: 2.5-7.5 mmol/24 hr).

In May 2025, as his adjusted calcium levels dipped to 1.89 mmol/L (reference range: 2.2-2.6 mmol/L) with a normal phosphate of 1.01 mmol/L (reference range: 0.81-1.5 mmol/L), his alfacalcidol was titrated to 1 µg three times a week on Mondays, Wednesdays, and Fridays, and he continued 750 ng for the remaining four days of the week. The patient was scheduled for follow-up in the endocrine clinic in six months for routine electrolyte review and medication optimization.

The patient was subsequently seen at the tertiary genetics center, where he reported worsening exertional dyspnea. Chest X-ray and echocardiogram did not show any abnormalities. An ophthalmology review was advised due to the risk of cataract development, and genetic counselling for his family was discussed. The biochemical findings for the patient, in chronological order of his visits, are summarized in Table 1.

**Table 1:** Chronological summary of biochemical parameters demonstrating persistent hypocalcemia and PTH elevation consistent with pseudohypoparathyroidism type 1b PTH: parathyroid hormone; eGFR: estimated glomerular filtration rate

Visit	September 2015	August 2023	November 2023	February 2024	April 2024	August 2024	November 2024	May 2025	Normal range
Calcium (mmol/L)	1.98	1.75	1.80	1.91	2.01	2.15	2.01	1.89	2.2-2.6 mmol/L
Phosphate (mmol/L)	1.70	1.58	1.49	1.71	1.51	1.34	1.71	1.01	0.81-1.50 mmol/L
Vitamin D (nmol/L)	86	92	101	Not tested	Not tested	Not tested	62	Not tested	50-174 nmol/L
PTH (pmol/L)	15.70	14.50	Not tested	12.20	Not tested	Not tested	12.00	7.50	1.1-6.9 pmol/L
eGFR (mL/min/1.73 m²)	64	44	54	55	53	47	49	51	>90 mL/min/1.73 m²

## Discussion

Lethargy is a commonly encountered non-specific symptom in primary care. A common cause to rule out is electrolyte imbalance. In this case report, the patient had untreated persistent hypocalcemia lasting over 10 years. The prevalence of hypocalcemia has previously been reported as high as 18% in all patients in the hospital and 85% in the intensive care unit [6]. Neuromuscular excitability, elicited in Chvostek's sign and Trousseau's sign, is one of the most common signs observed in hypocalcemia and can present as tetany in mild cases or seizures in more severe cases [7]. Of note, Chvostek's sign is not particularly sensitive for hypocalcemia (reported sensitivity 25%) compared to Trousseau's sign (reported sensitivity 94%); however, the reported specificity for Chvostek's sign is quite high at 96% [8,9]. 

**Figure 1:**
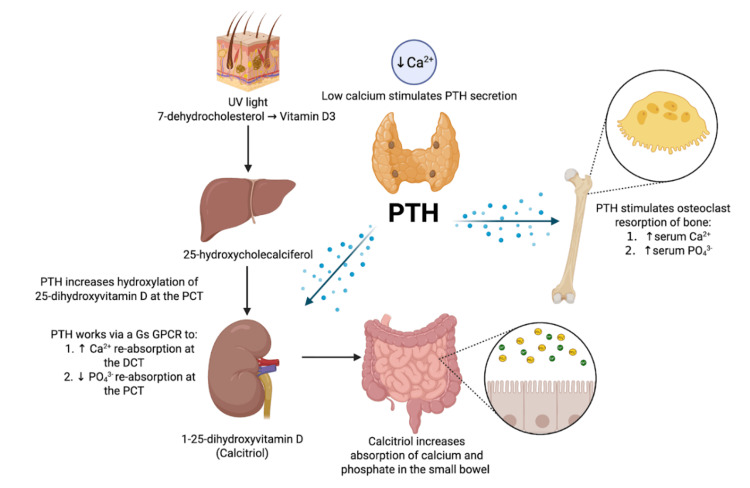
Calcium metabolism PTH: parathyroid hormone; PCT: proximal convoluted tubule; GPCR: G protein-coupled receptor; DCT: distal convoluted tubule The figure is an original creation of the authors, modified from [10].

Calcium physiology is complex; it is best approached from an understanding of the role of PTH in moderating calcium homeostasis. PTH acts on various organs, including the bone, intestine, and kidney. PTH receptors on organs mediate change via G protein-coupled receptors (GPCRs), using the Gs pathway to increase intracellular cAMP [4]. Salient features of calcium metabolism are summarized in Figure 1.

There are many causes of chronic hypocalcemia. Grossly, causes can be split into PTH-related and PTH-unrelated [2]. PTH-related causes include primary hypoparathyroidism (including iatrogenic/surgical, autoimmune), hypo-/hypermagnesemia, and PTH resistance. PTH-unrelated causes include renal insufficiency, vitamin D deficiency, and massive cellular lysis [7]. Vitamin D deficiency is one of the leading causes of chronic hypocalcemia in primary care, and its prevalence has been recorded as high as 50% [11].

Physiologically, PTH is produced in the chief cells of the parathyroid gland and is secreted into circulation to bind to PTH receptors at distant tissues. PTH receptors are GPCRs, inducing the dissociation of Gsα from the attached beta and gamma sub-units, thereby activating the alpha sub-unit to stimulate adenylyl cyclase, which converts adenosine triphosphate (ATP) to cyclic adenosine monophosphate (cAMP). cAMP has further downstream effects to include the activation of protein kinase A; the main outcome of interest is the modulation of serum calcium and phosphate based on which tissue is bound [4]. 

The term PHP was first used to describe a group of patients exhibiting a pattern of hypocalcemia and hyperphosphatemia with a lack of response to exogenous PTH extract in a case report by Albright and colleagues in 1942[12]. PHP should be suspected when the hormonal axis has been optimized through adequate oral calcium intake and vitamin D supplementation; however, downstream effects remain unobserved, i.e., suspicion should be raised for hormonal resistance. In this instance, we have observed persistent hypocalcemia, hyperphosphatemia, and elevated PTH despite vitamin D repletion.

**Figure 2:**
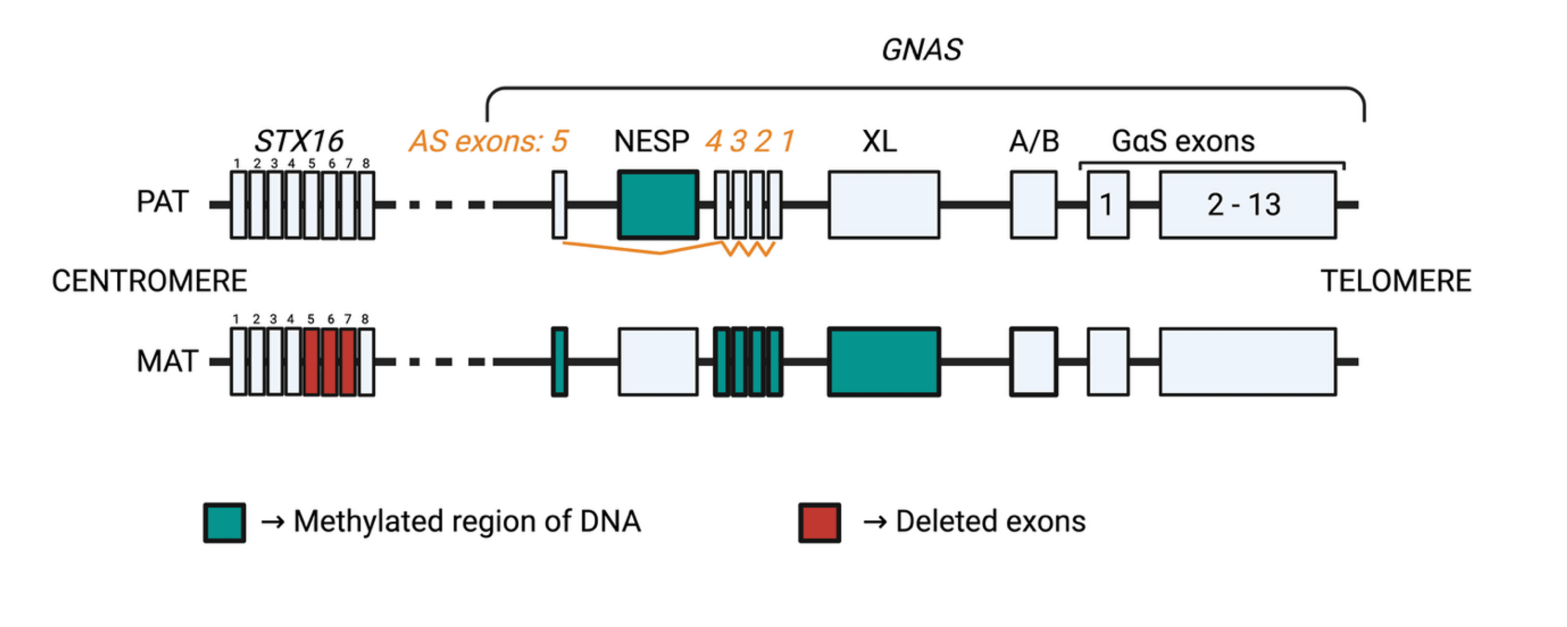
The GNAS locus in PHP1b PHP1b: pseudohypoparathyroidism type 1b The figure is an original creation of the authors, modified from [13].

The GNAS locus is a region of DNA on the q arm of chromosome 20 that encodes five main transcripts, of which four begin with promoters in germline differentially methylated regions (sequences of DNA which vary in methylation based on whether they are on the maternal or paternal allele, where methylation is established pre-fertilization (during gametogenesis)): NESP, AS, XL, and A/B. Any abnormality in methylation patterns for these exons (thought to be related to deletions in an upstream STX16 exon) can affect the expression of the fifth transcript of this locus, the GSα exon, on the maternal allele (the paternal allele is silenced via imprinting in the proximal convoluted tubule cells), resulting in an abnormality in the expression of GSα and subsequent dysfunction of the Gsα signalling pathway, ultimately resulting in PTH resistance [4,13]. A schematic model of the GNAS locus in PHP1b is demonstrated in Figure 2.

Similarly, GSα protein signalling is responsible for the mediation of other hypothalamic-pituitary axis hormones, including TSH and growth hormone. Resistance profiles for these hormones, in the presence of PTH resistance, should also raise suspicion for impairment of the Gs signalling pathway and the presence of PHP. 

The term PHP is an overarching diagnosis encompassing multiple subtypes: PHP1a, PHP1b, PHP1c, PPHP, and pseudohypoparathyroidism type 2 (PHP2). Across most bodily tissues, both maternal and paternal GSα are expressed [14]. Mutations or deletions of GSα exons may result in AHO. In contrast to PHP1b, which may involve deletions in STX16 and/or changes in methylation in maternal differentially methylated regions, PHP1a is a direct mutation or deletion in Gsα exons. PHP1a reflects the tissue-selective nature of imprinting; as the paternal GSα allele is silenced in proximal convoluted tubule cells, any mutations or deletions in the maternal allele will result in the impairment of PTH-mediated calcium homeostasis and phosphaturia. However, similar changes on the paternal Gsα allele would not cause issues with calcium or phosphate, but the morphological changes seen in AHO are present; this is termed PPHP. PHP1c shows a near-identical presentation to PHP1a, but there is expression of normal Gsα in erythrocytes in vitro. Patients with PHP2 demonstrate functioning Gsα and cAMP but do not have phosphaturia secondary to PTH; therefore, the suspected dysfunction resulting in hypocalcemia is thought to be further downstream of the GPCR [4,10]. 

It is pertinent that PHP1b can arise in either an autosomal dominant or sporadic inheritance pattern. The available literature explains that the STX16 or NESP exons may contain what are known as "imprinting control regions", which maintain differentially methylated regions in the GNAS locus. Deletions of the aforementioned exons may be associated with loss of Gsα expression [13]. The sporadic subtype is the most common; it features loss of methylation at A/B with variable loss of methylation at XL/AS and gain of methylation at NESP. 

In this case, the report confirmed an autosomal dominant maternally inherited pattern. This case emphasizes the importance of screening first-order female family members who are at risk of passing on the condition to offspring [13]. This patient did not demonstrate any AHO features; however, his serum biochemistry showed a pattern of PTH resistance with persistent low corrected calcium despite having normal serum vitamin D levels and no significant renal impairment. The possibility of PHP was considered, and subsequently, genetic testing was requested, which confirmed the diagnosis. Readers should also be aware of the broad range of genetic differentials to explain hypocalcemia, including DiGeorge syndrome and Barakat syndrome, although in contrast to PHP, these would not present with hyperparathyroidism [15].

With respect to management, the international consensus statement on PHP advises initiating active vitamin D. In PHP, the PTH axis is dysfunctional and blunts the renal 1α-hydroxylation of calcidiol (25-OH cholecalciferol) in the proximal convoluted tubules. Calcium supplementation and active vitamin D analogues, e.g., alfacalcidol, should be given under the regular monitoring of serum calcium and phosphate. The consensus statement advises that adjusted calcium levels should be targeted to the low-normal range. Nephrocalcinosis and nephrolithiasis can arise during the management of PHP as a result of increased urinary calcium excretion; therefore, it is advised to monitor 24-hour urinary calcium and to perform a renal ultrasound if nephrolithiasis is suspected. A low-phosphate diet can be advised. There is usually no role for phosphate binders in the long-term care of patients with PHP [5].

**Figure 3:**
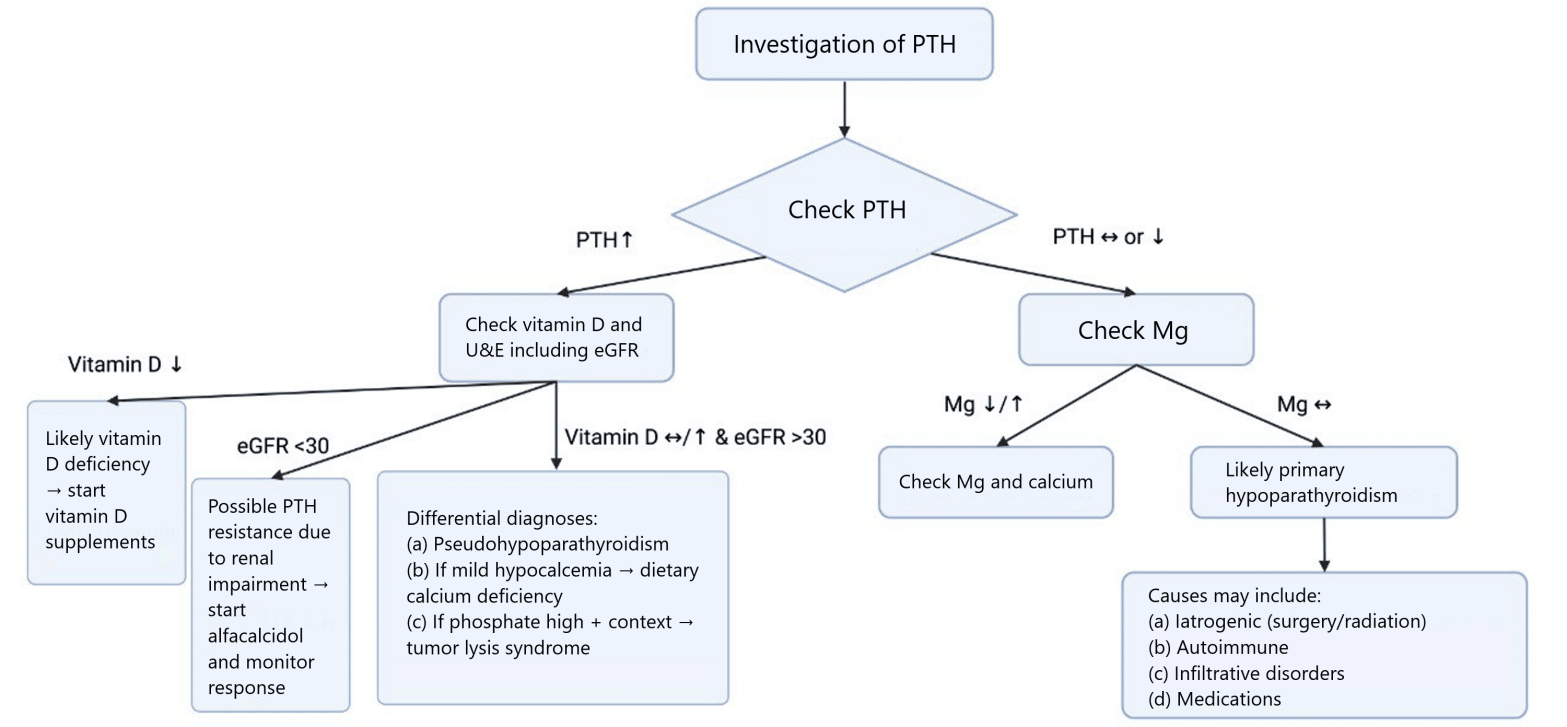
A structured approach to the investigation of hypocalcemia PTH: parathyroid hormone; U&E: urea and electrolytes; eGFR: estimated glomerular filtration rate; Mg: magnesium The figure is an original creation of the authors.

Recommended referrals include assessment by ophthalmology for posterior lens calcification, which can cause cataracts, and brain computed tomography (CT) imaging should be considered if any neurological abnormality is suspected due to the possible calcification of the basal ganglia [5]. Finally, family counselling is an essential step in the management plan, to offer genetic screening in first-degree relatives and to educate patients regarding the mode of inheritance, especially in type 1b, where female members give a 50% risk of transmission to offspring [5]. A systematic approach to the assessment of hypocalcemia can be summarized in Figure 3.

## Conclusions

This case emphasizes the importance of a structured approach in the diagnosis and management of patients presenting with hypocalcemia. PTH resistance is often explored in medical education in the context of pediatric presentations, almost always with a focus on AHO. It is atypical for a genetic diagnosis to be made for a patient in their 60s; however, readers are encouraged to seek an explanation even when patients may not be experiencing overt symptoms. Subtle features such as hypothyroidism and persistent nonresponsive hypocalcemia to medication should raise concerns of more rare causes, including PTH resistance, supported by the biochemical pattern, confirmed by genetic studies. In the United Kingdom, there is no formal pathway for the investigation of chronic hypocalcemia; however, we would suggest that such a pathway would help identify individuals who may benefit from further evaluation to prevent complications of mismanaged hypocalcemia and delayed diagnosis.
